# Supplementary material for: Development of Low-Grade Serous Ovarian Carcinoma from Benign Ovarian Serous Cystadenoma Cells
Source: Cancers (Basel). 2022 Mar 15;14(6):1506. doi: 10.3390/cancers14061506 (PMC8946187; doi:10.3390/cancers14061506)
Supplement: Supplementary file 1 [file cancers-14-01506-s001.zip › cancers-1601421-supplementary.pdf]

Article

Development of Low-Grade Serous Ovarian Carcinoma from Benign Ovarian Serous Cystadenoma Cells

Puja Dey[1], Kentaro Nakayama [1*], Sultana Razia [1], Masako Ishikawa [1], Tomoka Ishibashi [1], Hitomi Yamashita [1], Kosuke Kanno [1], Seiya Sato [1], Tohru Kiyono [2*] and Satoru Kyo [1]

Supplementary Table S1. Description of the primary antibodies.

Antibody	Manufacturer	Dilution Ratio	MW (kDa)
Anti-phospho-$p^{44/42}$MAPK	Cell Signaling (#4370)	1:2000 (WB)	42,44
Anti-$p^{44/42}$MAPK(Erk1/2)	Cell Signaling (#4695)	1:1000 (WB)	42.44
Anti-phospho-AKT (Ser473)	Cell Signaling (#4060)	1:2000 (WB)	60
Anti-AKT (pan)(C67E7)	Cell Signaling (#4691)	1:1000 (WB)	60
Anti-pan-Cytokeratin(C11)	Santa Cruz Biotechnology (sc-8018)	1:200 (WB)<br>1:50 (IHC)<br>1:100 (ICC)	40-59
GAPDH	Cell signaling (14C10)	1:1000 (WB)	37
Anti-PAX8	Proteintech (10336-1-AP)	1:50 (IHC)<br>1:100 (ICC)	
Anti-p-53	Santa Cruz Biotechnology (sc-47698)	1:50 (IHC)<br>1:100 (ICC)	
Anti-Estrogen Receptor alpha (SP1)	Invitrogen (#MA5-14501)	1: 200 (IHC)<br>1:100 (ICC)	
Anti-Progesterone Receptor (SP2)	Invitrogen (#MA5-14505)	1: 400 (IHC)<br>1:100 (ICC)	
Anti-Vimentin [EPR3776]	Abcam (ab92547)	1: 200 (IHC)<br>1: 250 (ICC)	

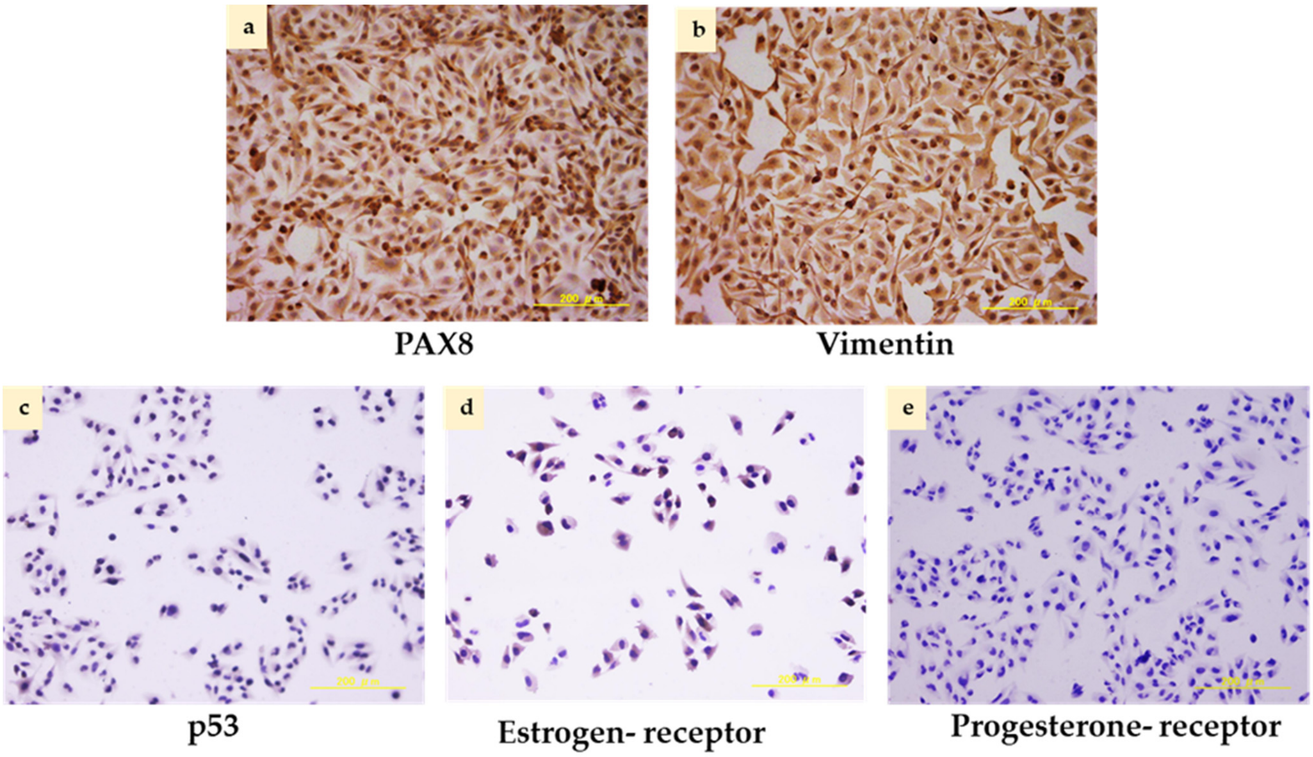

Supplementary Figure S1. Immunocytochemical analysis of HOVs-cyst-1 cells. The expression PAX8 (a), Vimentin (b), p53 (c), Estrogen (d) and Progesterone (e) receptor in immortalized HOVs-cyst-1 cells.

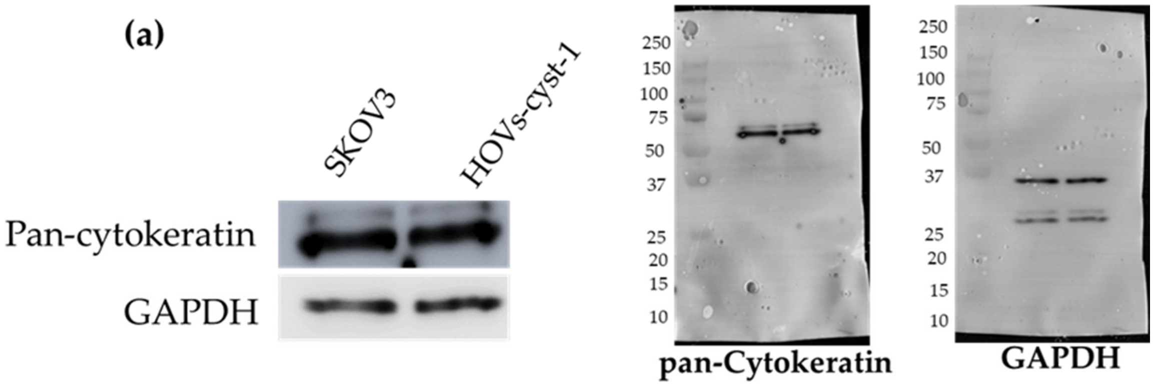

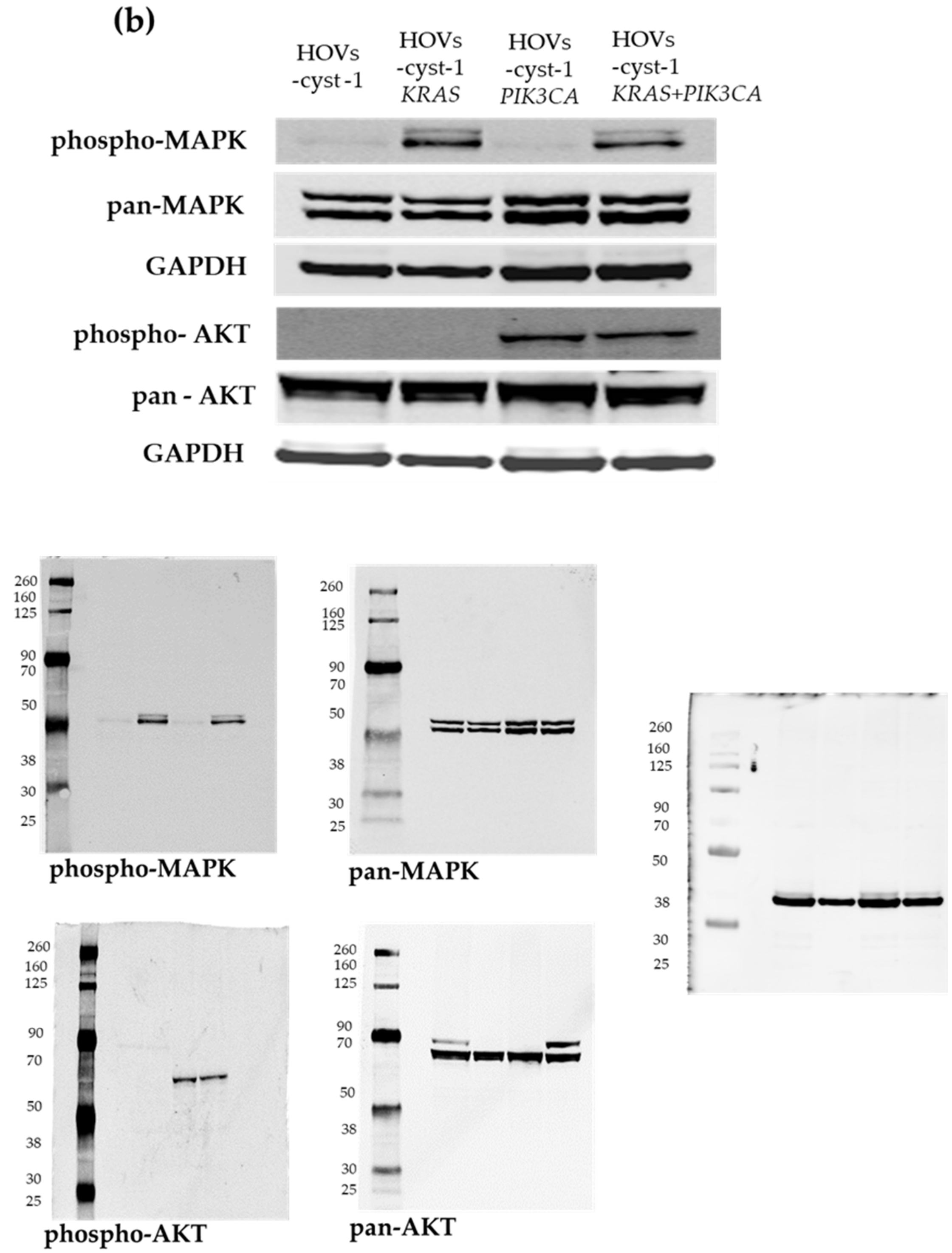

Supplementary Figure S2. (a) Western blot analysis of pan-Cytokeratin in immortalized HOVs-cyst-1 cells. **(b)** The expression of phospho-MAPK, pan-MAPK, phospho-AKT, pan-AKT expression in different transfectant cells.

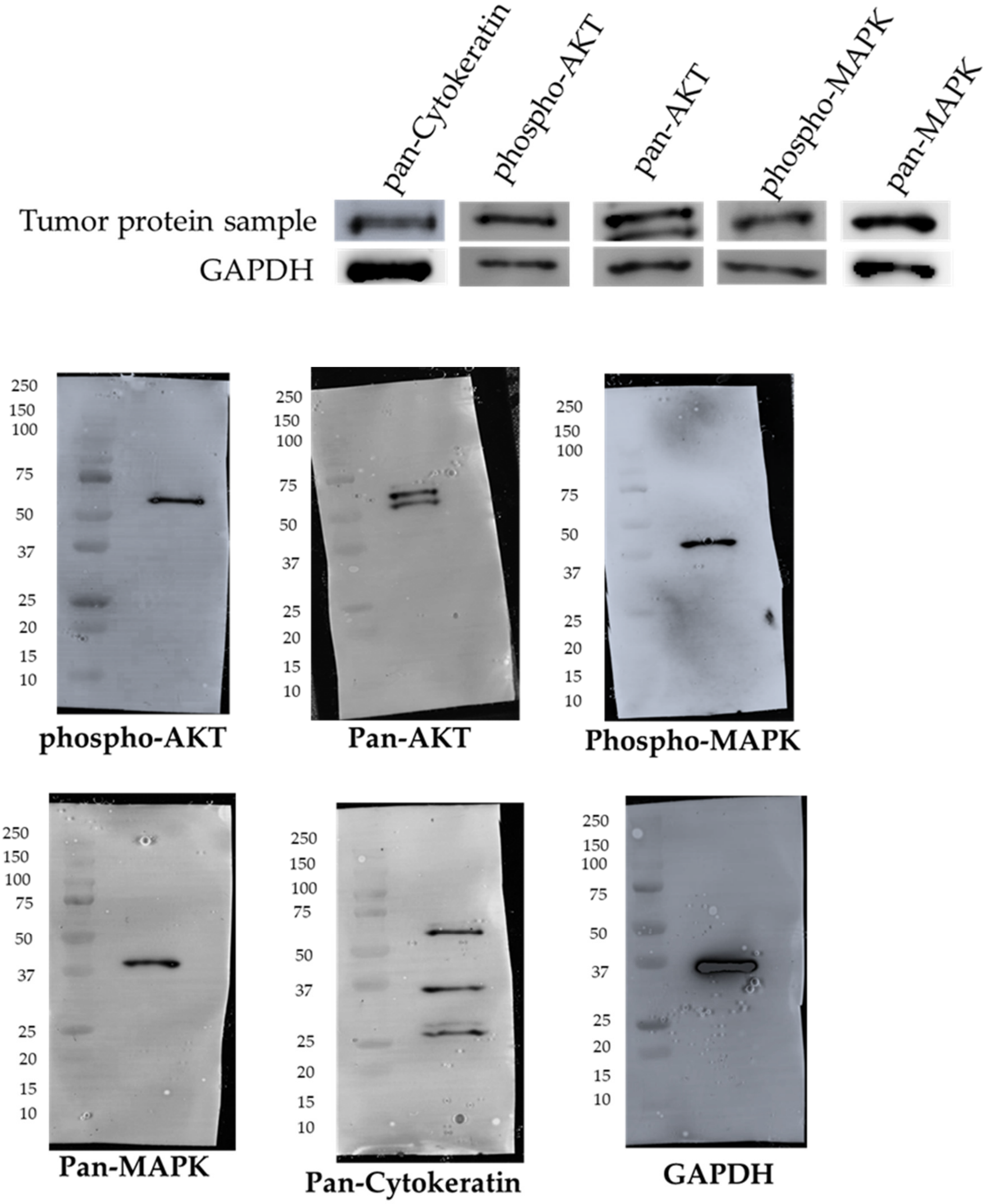

Supplementary Figure S3. Western blot analysis of xenograft tumors. Pan-Cytokeratin, phospho-AKT, pan-AKT, phospho-MAPK, and pan-MAPK expression levels were assessed via western blot analysis of HOVs-cyst-1 cells, with both *KRAS* and *PIK3CA* mutations, in intraperitoneal xenograft tumor. Activation of both RAS/ERK and PI3K/AKT signaling pathways confirmed by positive phospho-MAPK and phospho-AKT expression. Tumor protein loading

was determined using an antibody to the housekeeping gene, glyceraldehyde3-phosphate dehydrogenase (GAPDH, mouse monoclonal antibody).

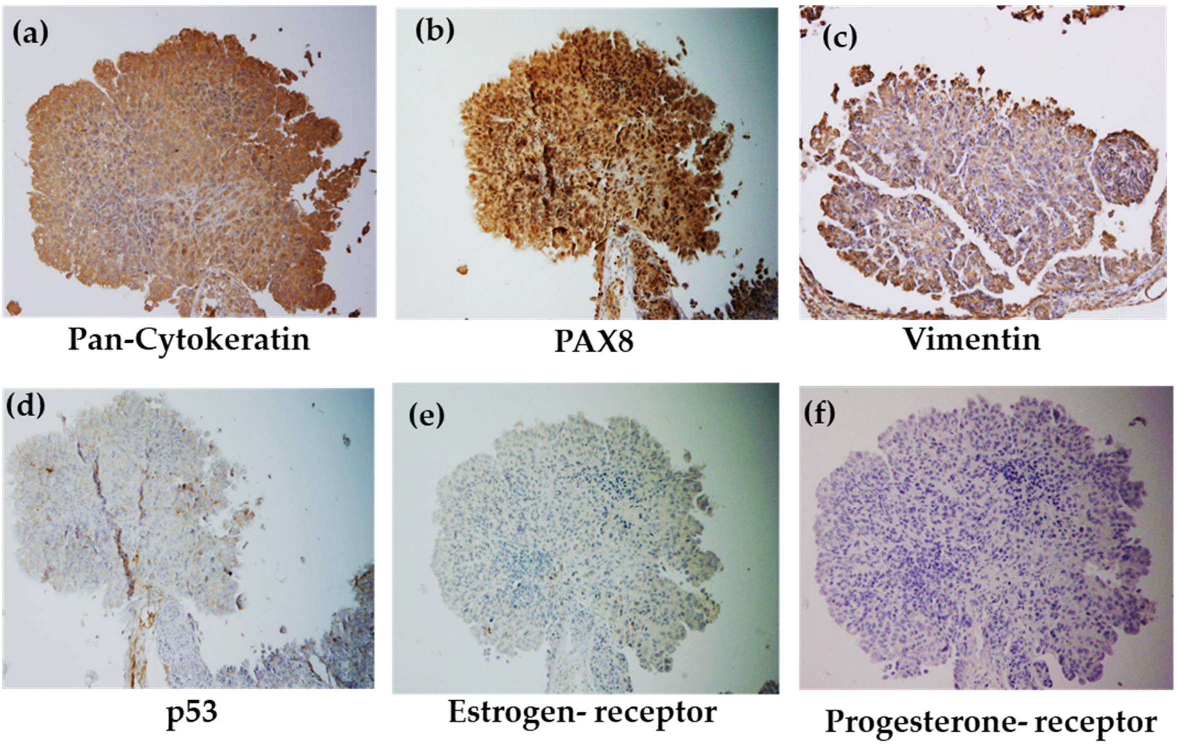

Supplementary Figure S4. Immunohistochemical analysis in xenograft tumors. The expression pattern of pan-Cytokeratin, PAX8, Vimentin, p53, Estrogen and Progesterone receptor (a, b, c, d, e, and f) in mouse xenograft tumors.

Supplementary Table S2. Whole exome sequencing results for HOVs-cyst-1.

Mutation type	Status
dMMR (deficient Mismatch Repair)	No mutation
HRD (Homologous recombinant Deficiency)	No mutation
OG (Oncogene)	No mutation
TSG (Tumor suppressor Gene)	No mutation
Other gene mutation	No mutation

Note: The whole exome sequencing reveals that there were no mutations present in HOVs-cyst-1 cells.

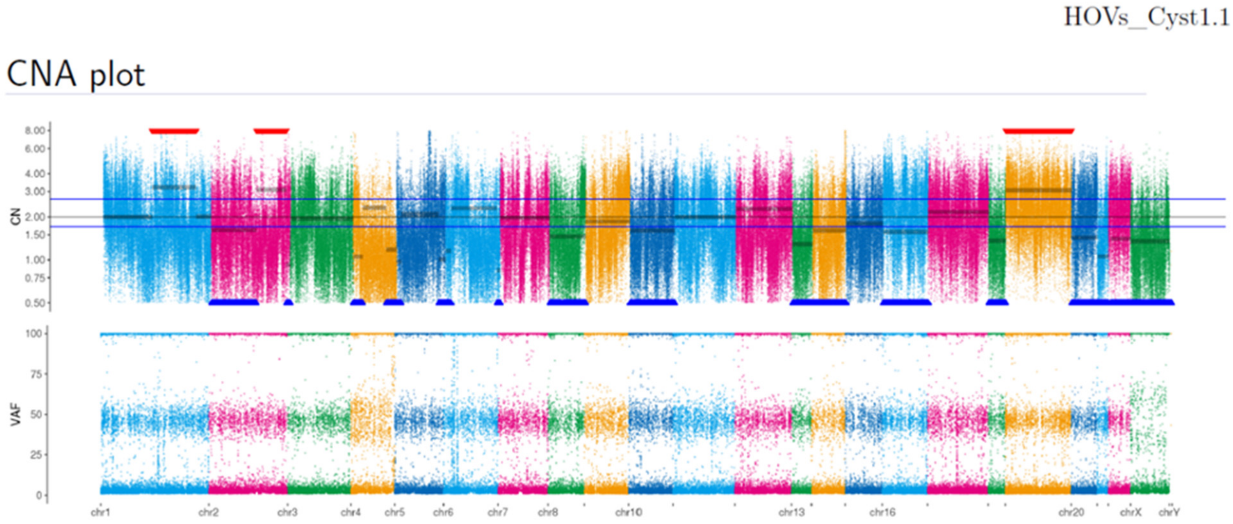

Supplementary Figure S5. Whole-exome sequencing showing no copy number alteration (CNA) in HOVs-cyst-1 cells.
